# *RUNX3* Methylation: An Epigenetic Biomarker for Early Liver Damage Induced by Co-Exposure to Aflatoxin B1 and Hepatitis B Virus

**DOI:** 10.3390/toxics13060425

**Published:** 2025-05-23

**Authors:** Yunying Mo, Xiaodan Lu, Shixiong Zheng, Junfeng Deng, Shihan Huang, Ye Hong, Xiaoyu Xian, Aliya Yijiati, Xingyu Yu, Xunwu Luo, Miner Xiao, Xingfen Yang, Michael N. Routledge, Yunyun Gong, Zhini He

**Affiliations:** 1 Food Safety and Health Research Center, School of Public Health, Southern Medical University, Guangzhou 510515, China; 2 The Sixth Affiliated Hospital of Jinan University, Dongguan 523000, China; 3 Leicester Medical School, University of Leicester, Leicester LE1 7RH, UK; 4 School of Food and Biological Engineering, Jiangsu University, Zhenjiang 212013, China; 5 School of Food Science and Nutrition, University of Leeds, Leeds LS2 9JT, UK

**Keywords:** AFB1, HBV, liver function, *RUNX3*, methylation

## Abstract

Aflatoxin B1 (AFB1), a well-established hepatic carcinogen, has limited research on early-stage epigenetic biomarkers for aflatoxin-induced liver damage. In this study, we investigated 168 unpackaged peanut oil (UPP) consumers to evaluate associations among AFB1 exposure, HBV infection, *RUNX3* methylation, and liver function. Our findings indicated an average daily AFB1 intake of 3.14 ng/kg·bw/day from UPP oil consumption. The high AFB1 exposure group exhibited significantly elevated gamma-glutamyl transferase (GGT) levels compared with the low AFB1 exposure group (*p* = 0.030). AFB1 exposure was negatively correlated with methylation status at the 2nd, 8th, and 9th CpG sites of *RUNX3* (r_s_ = −0.196, −0.192, −0.181, *p* = 0.021, 0.024, 0.036). Furthermore, methylation at the 8th and 9th CpG sites positively correlated with GGT (r_s_ = 0.206, 0.203, *p* = 0.019, 0.024). HBV infection significantly influenced *RUNX3* methylation, with the HBsAg^+^ group exhibiting 16.25% lower methylation (*p* < 0.05). Stratified analysis by HBV and AFB1 revealed that in the low AFB1 exposure subgroup, *RUNX3* methylation in the HBsAg^+^ group exhibited a significant 26.38% reduction compared with the HBsAg^−^ group. These results indicated that AFB1 and HBV independently and synergistically promote site-specific *RUNX3* hypomethylation. Our results implicated *RUNX3* methylation as a critical mediator in HBV-AFB1 co-exposure hepatotoxicity, potentially serving as a novel epigenetic biomarker for early liver damage detection.

## 1. Introduction

Aflatoxin B1 (AFB1), one of the most common and dangerous mycotoxins, is a Group I carcinogen classified by the International Agency for Research on Cancer (IARC) [1,2,3]. Its thermal stability and rapid growth rate in high-temperature, humid conditions like tropical and subtropical zones makes global grain contamination a serious issue [4,5,6]. Studies showed widespread AFT presence in cereals and their products globally, ranging from 0 to 1642 (µg/kg) for AFT detection in cereals and 0.002–1138.8 (µg/kg) for AFT detection in cereal products [7]. Our previous study revealed AFB1 detection in 79.72% of unpackaged peanut (UPP) oil samples (0.02 to 174.13 µg/kg), with a 60.00% exceedance rate in Southern China [8].

AFB1 primarily targets the liver in vivo, where it is metabolized into AFB1-DNA adducts, resulting in both acute toxicity and elevated hepatocellular carcinoma (HCC) risk [9]. Annually, 25,000–155,000 cases (about 3–20% of total HCC cases) were attributed to aflatoxins. Moreover, AFB1 exposure and HBV infection could synergistically increase the risk of HCC by 54-fold [10,11]. Given the high mortality rate of liver disease, preventing or slowing its progression has become the public health priority [12,13]. Despite advancements in HCC treatment, early symptoms are often subtle, leading to delayed detection and diagnosis typically occurring at advanced stages. This results in a poor 5-year survival rate of only about 18% [14,15,16]. Moreover, early-stage liver injury often presents as asymptomatic, and the currently available biomarkers for detecting liver diseases are still unable to fulfil the diagnostic standards for reliability, accuracy, and sensitivity [17]. Hence, it is crucial to have a deeper understanding of the molecular mechanisms of liver disease and to explore novel biomarkers for effective diagnosis and treatment, ultimately improving long-term survival.

DNA methylation is an epigenetic modification that maintains cellular homeostasis and influences disease development [18,19,20,21]. Notably, hypermethylation of the promoters in tumour suppressor genes is a significant epigenetic alteration observed in many cancers, including liver diseases [22,23,24,25]. Unlike genetic mutations, aberrant DNA methylation emerges early in liver disease (from fibrosis to cirrhosis to HCC), demonstrating substantial potential as an early detection biomarker [26,27,28]. Zhang et al. found that the methylated DNA could be detected years prior to the clinical diagnosis of HCC, indicating its importance in disease progression [29]. Moreover, AFB1 exposure has been linked to abnormal gene methylation in liver tissues, characterized by gene-specific hypermethylation (e.g., *RASSF1A*, *p16*, *GSTP1*, *MGMT*) and hypomethylation (e.g., *TXNRD1*, *PCNA*, *CCNK*, *RAB27A*, *HIST1H2BF*, and *DIAPH3*) [30,31,32,33,34], highlighting the potential of DNA methylation as a sensitive epigenetic biomarker for the early detection of AFB1-induced liver disease.

*RUNX3* is a multifunctional tumour suppressor gene located in chromosome 1p36-35, playing an important regulatory function in the essential processes of cell reproduction, differentiation, and apoptosis [35]. Emerging evidence indicates that *RUNX3* hypermethylation, frequently observed in various malignancies (e.g., bladder, lung, and colorectal cancers), leads to transcriptional silencing and contributes to tumour initiation and progression. This establishes *RUNX3* methylation as a potentially sensitive epigenetic biomarker for cancer detection [36,37,38,39]. Consistent with our previous findings, we observed *RUNX3* hypermethylation in 70% (14/20) of HCC cases. Interestingly, in vitro experiments revealed a contrasting pattern; short-term AFB1 exposure reduced *RUNX3* methylation levels in L02R cells, which contrasts with the hypermethylation status observed in HCC tissues [40]. These differential methylation patterns suggest that *RUNX3* methylation is dynamically associated with liver disease progression. However, the potential of *RUNX3* methylation as an early epigenetic biomarker for AFB1-induced hepatocarcinogenesis remains to be fully elucidated. To address this knowledge gap, we investigated *RUNX3* methylation levels in a cohort exposed to both AFB1 and HBV, aiming to evaluate its potential as a sensitive biomarker for monitoring early liver disease caused by co-exposure to AFB1 and HBV.

## 2. Materials and Methods

### 2.1. Subjects Recruitment

This study initially enrolled 168 volunteers through a standardized recruitment process. After excluding participants with incomplete biospecimen data, the final cohort comprised 150 participants who were eligible for subsequent analyses. The detailed recruitment criteria and questionnaire specifications have been previously documented [8]. In brief, we administered structured questionnaires to collect comprehensive participant information, encompassing demographic characteristics, medical history, and dietary patterns. Additionally, all the blood samples of the participants were obtained for the assessment of liver function parameters and HBV infection status. The study protocol was approved ethically by the Institutional Review Board of the 5th Hospital of Southern Medical University (approval no.: 2019-YYK-004).

### 2.2. Aflatoxins Exposure Assessment

In all, 114 UPP oil samples were collected and analysed for aflatoxin B1 (AFB1), aflatoxin B2 (AFB2), aflatoxin G1 (AFG1), and aflatoxin G2 (AFG2) by using high-performance liquid chromatography tandem mass spectrometry (HPLC-MS/MS), following the Chinese National Standards (National Standard for Food Safety: Determination of Aflatoxins Group B and G in Foods, GB 5009.22-2016) [41]. The analytical methodology was implemented as previously described [8]. Subsequently, we evaluated the estimated daily intake (EDI, ng/kg·bw/day) of AFB1 from UPP oil consumption for all study participants using the following formula:

AFB1 EDI = Consumption of peanut oil (g/d) × Contamination of AFB1 in peanut oil (μg/kg)/bw (kg)

### 2.3. Liver Function and HBV Infection Detection

Liver function was evaluated using an automatic biochemistry analyser (BS-240VET, Mindray Bio-Medical Electronics Ltd., Shenzhen, China). The comprehensive panel for liver function assessment included the following seven key indicators: aspartate aminotransferase (AST), alanine aminotransferase (ALT), alkaline phosphatase (ALP), gamma-glutamyl transpeptidase (GGT), total serum protein (TP), total bilirubin (TBIL), and albumin (ALB). The status of HBV infection was determined by combining serological markers and molecular tests, which included hepatitis B surface antigen (HBsAg), hepatitis B e antigen (HBeAg), hepatitis B surface antibody (anti-HBs), hepatitis B e antibody (anti-HBe), hepatitis B core antibody (anti-HBc), and quantitative assays for HBV DNA.

### 2.4. Pyrosequencing for DNA Methylation Detection

**Figure A1:**
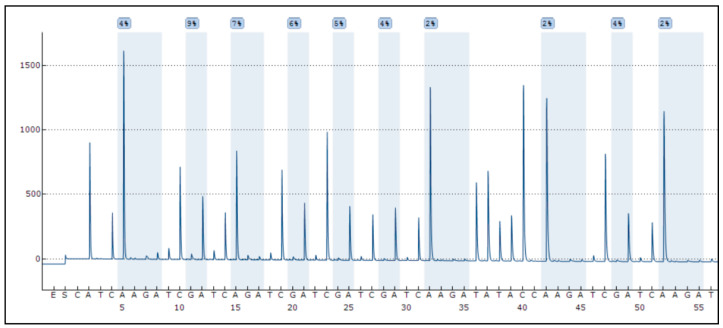
The target region for pyrosequencing indicates the target fragments (−436 bp, −364 bp).

The methylation levels of *RUNX3* in peripheral blood samples from study participants were quantitatively analysed using bisulphite sequencing PCR (BSP), with methodological details previously described [40]. Based on BSP analysis, we identified the significantly altered regions as the target fragments for subsequent pyrosequencing analysis (−436 bp, −364 bp), which contain a total of nine CpG sites. These sites are sequentially named CpG site 1 to 9. The mean methylation levels across the nine CpG sites were calculated as the methylation level of *RUNX3* (Figure A1).

Pyrosequencing analysis was conducted following established protocols [40,42,43,44]. Briefly, 1 µg genomic DNA was subjected to bisulphite conversion using the EpiTect Bisulfite Kit (Qiagen, Hilden, Germany) according to the manufacturer’s protocol. Subsequently, 50 ng bisulphite modified DNA was amplified into a 25 µL PCR reaction mixture. After verifying the specificity of PCR product, they were then analysed using the PyroMark Q24 system (Qiagen, Germany) for pyrosequencing-based quantitative methylation analysis.

### 2.5. Statistical Analysis

All continuous variants were evaluated for normality prior to the statistical analyses. Parametric tests (Student’s *t*-test and Chi-square test) were used to analyse variables demonstrating normal distribution, while a non-parametric method (Mann–Whitney U test) was used to analyse non-normally distributed variables. Bivariate correlations were assessed through linear regression analysis. The intrinsic relationships among variables were assessed through factor analysis. The results were considered statistically significant with *p* < 0.05. The SPSS statistical software (version 26.0) was performed for all statistical analyses.

## 3. Results

### 3.1. Demographic Information of the Study Population

The participants in this study were divided into low AFB1 exposure (n = 75) and high AFB1 exposure groups (n = 75) based on the median AFB1 estimated daily intake (EDI) level (3.14 ng/kg·bw/day). The preliminary analysis indicated no significant differences between groups in terms of age, body mass index (BMI), waist-to-hip ratio, or gender (Table 1). However, significant differences were observed in smoking, drinking status, and educational attainment (*p* = 0.001, 0.040, 0.026, respectively). Specifically, both tobacco and alcohol use were significantly more common in the high AFB1 exposure group compared with the low AFB1 exposure group. The prevalence of current or former smokers was 25.3% higher, and the prevalence of current or former drinkers was 18.7% higher in the high AFB1 exposure group compared with the low AFB1 exposure group.

### 3.2. Effect of AFB1 Exposure on Liver Function and RUNX3 Methylation

We evaluated liver function and *RUNX3* methylation levels in the peripheral blood of both groups. Our analysis revealed a significant elevation in gamma-glutamyl transferase (GGT) levels in the high AFB1 exposure group compared with the low AFB1 exposure group (*p* = 0.030). Although the methylation levels of *RUNX3* showed a general decreasing trend in the high AFB1 exposure group, these variations failed to reach statistical significance (Table 2).

Previous studies have shown a significant impact of HBV on AFB1-induced hepatotoxicity, in order to have a more precise analysis on the independent effects of AFB1 exposure for liver function and *RUNX3* methylation; thereafter, we excluded HBV infection, the important confounding factor in the subsequent analyses. We divided the remaining HBsAg-negative participants (HBsAg^−^) into low AFB1 exposure (n = 65) and high AFB1 exposure (n = 62) groups based on the median AFB1 EDI (2.86 ng/kg·bw/day). Within the HBsAg^−^ population, the methylation levels of the 2nd, 8th, and 9th CpG sites of *RUNX3* were significantly reduced in the high-exposure group, with decreases of 20.03%, 4.83%, and 7.69%, respectively (*p* = 0.044, 0.033, 0.037) (Table 3). However, after excluding the HBsAg^+^ individuals, the previously observed difference in GGT levels between the exposure groups became statistically insignificant. These results demonstrate that AFB1 exposure can alter *RUNX3* methylation levels, while also highlighting the critical role of HBV infection in modulating AFB1-mediated hepatotoxicity.

### 3.3. Effect of HBV Infection on Liver Function and RUNX3 Methylation Levels

Considering the established effects on liver function associated with HBV infection, we hypothesized that HBV might also influence *RUNX3* methylation status. To test this hypothesis, we conducted a comprehensive analysis of HBV infection on both liver function and *RUNX3* methylation patterns (Table 4). The population was stratified according to HBV infection status into HBsAg^+^ group (n = 23) and HBsAg^−^ group (n = 127). The results showed that AST and ALT levels were significantly higher in the HBsAg^+^ group (*p* = 0.012, 0.014, respectively), with increases of 1.29-fold and 1.41-fold, respectively. Conversely, TBIL levels were reduced by 13.56% in the HBsAg^+^ group (*p* = 0.042). Notably, *RUNX3* methylation levels significantly reduced by 16.25% (6.03 vs. 5.05, *p* < 0.05) in the HBsAg^+^ group, with decreases of 21.31%, 25.37%, 16.51%, 10.79%, 8.75%, 9.45%, 15.91%, 21.37%, and 19.13% for CpG sites 1 to 9, respectively (all the *p* < 0.05). Collectively, these findings suggest that HBV infection has a more pronounced influence on *RUNX3* methylation patterns than AFB1 exposure.

Furthermore, we conducted stratified analysis in accordance with AFB1 exposure and HBV infection. The results (Table A1) demonstrated significant differences in the methylation levels of *RUNX3* between HBsAg^+^ and HBsAg^−^ groups within the low AFB1 exposure subgroup (all *p* < 0.05). Specifically, the HBsAg^+^ group exhibited significantly lower methylation levels of *RUNX3* compared with the HBsAg^−^ group, with an average reduction of 26.38% (4.55 vs. 6.18, *p* < 0.05). Additionally, in the HBsAg^−^ subgroup analysis, we observed a significant 16.57% decrease in methylation levels at the 2nd CpG site in the high AFB1 exposure group (*p* = 0.044). These findings indicate that both AFB1 exposure and HBV infection may contribute to the site-specific hypomethylation of *RUNX3*.

### 3.4. Correlation Analysis of RUNX3 Methylation Levels with AFB1 Exposure and HBV Infection

**Figure A2:**
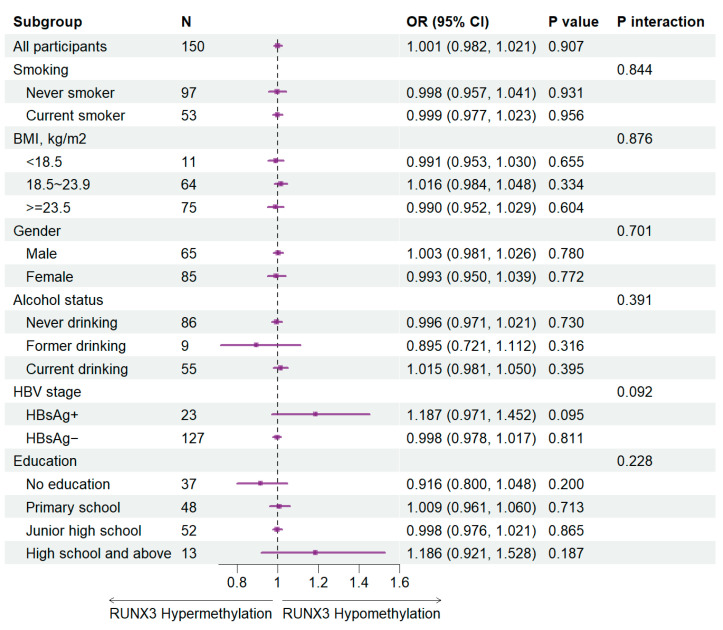
Subgroup analysis of AFB1 exposure and *RUNX3* methylation levels forest map.

We conducted a correlation analysis between AFB1 exposure and various parameters, including liver function indicators and *RUNX3* methylation levels. To evaluate potential confounding factors, we initially performed subgroup analyses (Figure A2), which revealed no significant differences in BMI, gender, smoking, drinking status, or educational attainment between the comparison groups. Subsequent analysis indicated no significant association between AFB1 exposure and liver function (Table 5). However, there was a negative correlation between AFB1 exposure and the methylation status at 2nd, 8th and 9th CpG sites of *RUNX3* (r_s_ = −0.196, −0.192, −0.181, all the *p* < 0.05). Further analysis explored the relationship between the methylation levels of these specific CpG sites (2nd, 8th, and 9th) and liver function. The results showed positive correlations between GGT levels and methylation status at the 8th and 9th sites (r_s_ = 0.206, 0.203, both *p* < 0.05) (Table 6).

Additionally, we examined the correlation between AFB1 exposure and *RUNX3* methylation in the HBsAg^−^ population. Our findings (Table A2) demonstrated that the methylation levels at multiple *RUNX3* CpG sites, including the 1st, 2nd, 3rd, 4th, 8th, and 9th positions were all negatively correlated with AFB1 exposure (r_s_ = −0.192, −0.241, −0.207, −0.180, −0.288, −0.295, all *p* < 0.05). Notably, the methylation levels at the 8th and 9th CpG sites showed significant negative correlations with ALP (r_s_ = −0.209, −0.192, both *p* < 0.05) (Table A3). These findings collectively demonstrated that both AFB1 exposure and HBV infection are associated with the site-specific hypomethylation of *RUNX3*, indicating a potential synergistic effect on epigenetic regulation.

### 3.5. Factorial Analysis of Methylation Levels of RUNX3 with AFB1 Exposure and HBV Infection

The above results indicated that both AFB1 exposure and HBV infection could induce *RUNX3* hypomethylation in a site-specific manner. To further elucidate the potential synergistic effects between these two factors, we performed a comprehensive factorial analysis to examine their combined impacts of *RUNX3* methylation levels. The results revealed significant interaction effects between HBV infection and AFB1 exposure at the 5th, 8th, and 9th CpG site of *RUNX3* (all the *p* < 0.05). Remarkably, AFB1 exposure exhibited a significant main effect specifically at the 5th CpG site. In contrast, HBV infection exhibited significant main effects at multiple CpG sites, including the 1st, 2nd, 3rd, 4th, and 7th CpG sites (all the *p* < 0.05), suggesting a more extensive influence on *RUNX3* methylation patterns (Table A4). In summary, these findings suggest that HBV infection has a more substantial and widespread impact on *RUNX3* methylation compared with AFB1 exposure.

## 4. Discussion

Emerging evidence indicates that DNA methylation alterations play crucial roles in liver disease progression, and their reversible nature makes them promising epigenetic biomarkers for early diagnosis and targeted therapies [45,46,47]. Our study reveals that both AFB1 exposure and HBV infection can induce the site-specific hypomethylation of *RUNX3*, suggesting its potential as a novel epigenetic biomarker of co-exposure to AFB1 and HBV. In addition, we found that HBV infection exhibits a more substantial and widespread impact on *RUNX3* methylation compared with AFB1 exposure, which indicates that *RUNX3* could be taken into consideration, while exploring the mechanisms underlying HBV induced hepatotoxicity.

In our previous investigation [8], we demonstrated a synergistic hepatotoxic effect between HBV infection and AFB1 exposure, wherein elevated aflatoxin exposure exacerbated HBV-induced liver dysfunction. In this study, we grouped the participants into two groups by the median AFB1 exposure level and found that the GGT was significantly higher in the high AFB1 exposure group than the low AFB1 exposure group; although, this association diminished after excluding the HBsAg^+^ population. Subsequent stratification by HBV infection revealed significantly elevated AST and ALT levels in the HBsAg^+^ group compared with the HBsAg^−^ group; whereas, TBIL levels were notably reduced in the HBsAg^+^ group. Previous studies have demonstrated that HBsAg^+^ patients exhibited significantly elevated AST and TBIL levels alongside reduced ALB concentrations [48], with HBV DNA-positive patients showing particularly increased ALT activity [49]. These findings suggested distinct hepatotoxic mechanisms between HBV infection and AFB1 exposure, potentially through the differential modulation of hepatic metabolic enzymes, which is consistent with our results. However, the precise underlying mechanisms require further elucidation.

In the pathological progression of liver diseases, DNA methylation modifications exert pivotal regulatory functions [50]. Extensive research has demonstrated that both AFB1 exposure and HBV infection significantly influence gene-specific methylation alterations in hepatic tissues [51,52,53]. Additionally, the synergistic effects of AFB1 and HBV can induce aberrant changes in the DNA methylation of critical tumour suppressor genes, including *p53* and *PTEN*, thereby exacerbating liver injury [54,55]. Studies have demonstrated that *RUNX3* was significantly hypermethylated in HBV-related HCC [56], while during AFB1-induced hepatocytes’ malignant transformation, *RUNX3* exhibited a dynamic DNA methylation shift, transitioning from initial hypomethylation to hypermethylation [40]. These indicated that during the progression of liver diseases, both AFB1 exposure and HBV infection could influence the methylation status of *RUNX3*. The findings were in agreement with our results, which indicated that in the HBsAg^−^ population, AFB1 exposure induced the *RUNX3* hypomethylation in a site-specific manner, particularly at the 2nd, 8th, and 9th CpG sites in the high AFB1 exposure group. When grouping the participants by HBV infection status (HBsAg^+/−^), we observed significant differences in *RUNX3* methylation, with a 16.25% reduction in the HBsAg^+^ group. Unfortunately, in the overall population, a non-significant trend of reduced *RUNX3* methylation levels was observed in the high AFB1 exposure group compared with the low-exposure group.

Furthermore, according to the correlation analysis, we found that AFB1 exposure induces the aberrant methylation of *RUNX3* in HBsAg^+^ participants. In the low AFB1 exposure subgroup, *RUNX3* showed significant hypomethylation (reduced by 26.38%) in the peripheral blood of HBsAg^+^ patients. This suggests potential interactions between HBV infection and AFB1 exposure to *RUNX3* methylation. To further clarify this issue, we conducted a factorial analysis. The results demonstrated that while both factors jointly influenced the 5th, 8th, and 9th CpG sites of *RUNX3*, AFB1 predominantly affected the 5th CpG site, and HBV significantly modulated the 1st, 2nd, 3rd, 4th, and 7th CpG sites. In summary, these findings indicated that both AFB1 exposure and HBV infection could induce *RUNX3* hypomethylation in a site-specific manner, with a greater impact of HBV, suggesting that *RUNX3* could be considered as a potential target in future studies on the mechanisms of co-exposure to AFB1- and HBV-induced toxic effects.

Epidemiological studies have demonstrated that in regions showing the co-exposure of AFB1 and HBV, such as southern China and Africa, the synergistic effects of AFB1 and HBV can alter the expression of specific genes, which are significantly associated with alterations in hepatic metabolic enzymes. For instance, co-exposure to AFB1 and HBV can induce mutations in the *p53* gene [57,58], leading to hepatocellular dysfunction and subsequent alterations in hepatic metabolic enzymes levels. However, systematic research on the relationship between gene alterations and hepatic metabolic enzymes under the combined effects of AFB1 and HBV remains limited. As mentioned previously, our research has confirmed that HBV infection and AFB1 exposure contribute to liver injury through distinct hepatotoxic mechanisms, primarily by regulating the activity of different hepatic metabolic enzymes. Additionally, both AFB1 exposure and HBV infection could induce *RUNX3* hypomethylation in a site-specific manner. To further investigate these relationships, we analysed the correlation between *RUNX3* methylation levels and the following three key factors: AFB1 exposure, HBV infection, and liver function. Our analysis demonstrated a significant negative correlation between the methylation levels at the 2nd, 8th, and 9th CpG sites of *RUNX3* and AFB1 exposure levels. Furthermore, we observed a distinct positive correlation between the methylation levels of the 8th and 9th sites and serum GGT levels. Besides, in the HBsAg^−^ population, we observed that the methylation levels of the 1st, 2nd, 3rd, 4th, 8th, and 9th CpG sites in *RUNX3* are negatively correlated with AFB1 exposure, and the methylation levels at the 8th and 9th CpG sites of *RUNX3* demonstrated an additional negative correlation with serum ALP levels. These findings collectively suggested that both AFB1 exposure and HBV are associated with the site-specific hypomethylation of *RUNX3*. Importantly, our data indicated that GGT levels, as a liver function parameter influenced by AFB1-HBV co-exposure, may be particularly associated with the methylation status of the 8th and 9th CpG sites.

In conclusion, our study demonstrated that both AFB1 exposure and HBV infection may contribute to the site-specific hypomethylation of *RUNX3*, implicating *RUNX3* methylation as a critical mediator in the hepatotoxicity caused by co-exposure to HBV and AFB1, potentially serving as a novel epigenetic biomarker for early liver damage detection. Furthermore, the alterations in liver function parameters induced by AFB1-HBV co-exposure exhibited a significant correlation with *RUNX3* methylation patterns; although, the potential of the molecular mechanism remains to be clarified and warrants further investigation.

The relatively limited samples collected in this study may introduce potential biases. In addition, this study investigated the methylation alteration of *RUNX3* in the population consuming UPP oil, focusing on the dose–effect relationship between AFB1 exposure and HBV infection on the methylation of *RUNX3*. However, the study failed to include the changes in *RUNX3* methylation in the population without AFB1 exposure, and this is one of the limitations of this study.

## Figures and Tables

**Table 1:** Demographic information of population in this study.

	Low AFB1 Exposure (n = 75)	High AFB1 Exposure (n = 75)	*p*
	Mean ± SD	Mean ± SD	
Age (year)	49 ± 8.90	49 ± 9.59	0.665
BMI	23.26 ± 3.34	23.60 ± 3.53	0.754
Waist hip ratio	0.91 ± 0.05	0.92 ± 0.05	0.637
Gender			0.485
Female	51 (68%)	34 (45.3%)	
Male	24 (32%)	41 (54.7%)	
Smoking status			0.001 *
Never smoker (Y, %)	58 (77.3%)	39 (52%)	
Former and Current smoker (Y, %)	17 (22.7%)	36 (48%)	
Drinking status			0.040 *
Never drinking (Y, %)	50 (66.7%)	36 (48%)	
Former and Current drinking (Y, %)	25 (33.3%)	39 (52%)	
Education			0.026 *
No education (Y, %)	21 (28%)	16 (21.3%)	
Primary school (Y, %)	31 (41.3%)	17 (22.7%)	
Junior high school (Y, %)	16 (21.3%)	36 (48%)	
High school and above (Y, %)	7 (9.3%)	6 (8%)	

Kolmogorov–Smirnov was used for normal distribution test. Student *t*-test and Mann–Whitney U test was used for continuous variables, Chi-square test was used for category variables statistical analysis. * *p* < 0.05.

**Table 2:** Liver functions and *RUNX3* methylation status in low/high AFB1 exposure groups.

	Low AFB1 Exposure (n = 75)	High AFB1 Exposure (n = 75)	*p*
	Mean ± SD	Mean ± SD	
AFB1 EDI (ng/kg BW/d)	0.62 ± 0.90	18.35 ± 20.60	<0.001 *
AFT EDI (ng/kg BW/d)	1.01 ± 1.60	21.22 ± 24.35	<0.001 *
Liver function and lipid metabolism			
AST (U/L)	22.69 ± 7.23	24.00 ± 8.71	0.506
ALT (U/L)	21.83 ± 15.16	24.03 ± 14.67	0.507
ALP (U/L)	82.39 ± 22.21	89.01 ± 24.46	0.162
GGT (U/L)	30.59 ± 23.98	43.52 ± 43.59	0.030 *
TP (g/L)	77.30 ± 3.58	76.65 ± 4.00	0.430
TBIL (μmol/L)	10.06 ± 5.30	10.31 ± 4.41	0.314
ALB (g/L)	47.74 ± 1.84	47.75 ± 2.37	0.749
*RUNX3* methylation			
*RUNX3* site1	6.34 ± 3.44	6.09 ± 3.29	0.959
*RUNX3* site2	12.57 ± 5.69	10.84 ± 4.77	0.116
*RUNX3* site3	8.59 ± 2.66	8.43 ± 2.26	0.838
*RUNX3* site4	7.10 ± 1.56	7.14 ± 1.51	0.979
*RUNX3* site5	4.95 ± 0.82	4.99 ± 0.90	0.882
*RUNX3* site6	4.54 ± 0.61	4.84 ± 1.96	0.582
*RUNX3* site7	2.23 ± 1.11	2.08 ± 0.56	0.826
*RUNX3* site8	2.74 ± 1.40	2.31 ± 0.95	0.444
*RUNX3* site9	5.24 ± 2.31	4.60 ± 1.53	0.462
*RUNX3* Total	5.96 ± 2.04	5.81 ± 1.73	0.967

Mann–Whitney test was used here. * *p* < 0.05.

**Table 3:** Liver functions and *RUNX3* methylation in different AFB1 exposure groups in HBsAg^−^ population.

	Low AFB1 Exposure (n = 65)	High AFB1 Exposure (n = 62)	*p*
	Mean ± SD	Mean ± SD	
AFB1 EDI (ng/kg BW/d)	0.01 ± 0.90	12.64 ± 22.10	<0.001 *
AFT EDI (ng/kg BW/d)	0.09 ± 1.68	14.81 ± 26.23	<0.001 *
Liver function and lipid metabolism			
AST (U/L)	22.00 ± 6.46	21.00 ± 6.34	0.898
ALT (U/L)	18.00 ± 14.74	18.00 ± 12.76	0.990
ALP (U/L)	82.00 ± 21.87	84.50 ± 24.69	0.368
GGT (U/L)	23.00 ± 24.01	28.00 ± 43.91	0.165
TP (g/L)	77.50 ± 3.73	77.25 ± 4.05	0.561
TBIL (μmol/L)	8.90 ± 4.85	10.75 ± 4.48	0.089
ALB (g/L)	47.65 ± 1.87	47.90 ± 1.96	0.186
*RUNX3* methylation			
*RUNX3* site1	5.18 ± 3.52	4.52 ± 3.33	0.175
*RUNX3* site2	12.43 ± 5.73	9.94 ± 4.97	0.044 *
*RUNX3* site3	8.68 ± 2.63	7.93 ± 2.56	0.142
*RUNX3* site4	7.33 ± 1.52	6.95 ± 1.67	0.191
*RUNX3* site5	5.06 ± 0.76	4.96 ± 0.97	0.300
*RUNX3* site6	4.39 ± 0.63	4.52 ± 2.11	0.950
*RUNX3* site7	2.00 ± 1.15	1.82 ± 0.59	0.241
*RUNX3* site8	2.07 ± 1.43	1.97 ± 1.00	0.033 *
*RUNX3* site9	4.29 ± 2.36	3.96 ± 1.58	0.037 *
*RUNX3* Total	5.70 ± 2.04	5.32 ± 1.87	0.195

Mann–Whitney test was used here. * *p* < 0.05.

**Table 4:** Liver functions and *RUNX3* methylation levels in HBsAg^+/−^ groups.

	HBsAg^−^ (n = 127)	HBsAg^+^ (n = 23)	*p*
	mean ± SD	mean ± SD	
AFB1 EDI (ng/kg BW/d)	10.21 ± 18.28	5.49 ± 5.57	0.960
AFT EDI (ng/kg BW/d)	11.91 ± 21.43	6.73 ± 6.74	0.990
Liver function and lipid metabolism			
AST (U/L)	22.35 ± 6.37	28.87 ± 12.80	0.012 *
ALT (U/L)	21.56 ± 13.76	30.48 ± 18.69	0.014 *
ALP (U/L)	85.62 ± 23.39	86.13 ± 24.76	0.902
GGT (U/L)	36.10 ± 35.54	42.38 ± 36.61	0.417
TP (g/L)	77.15 ± 3.89	76.02 ± 3.16	0.218
TBIL (μmol/L)	10.40 ± 4.68	8.99 ± 5.73	0.042 *
ALB (g/L)	47.92 ± 1.92	46.73 ± 2.91	0.061
*RUNX3* methylation			
*RUNX3* site1	6.43 ± 3.36	5.06 ± 3.18	0.036 *
*RUNX3* site2	12.14 ± 5.46	9.06 ± 3.23	0.020 *
*RUNX3* site3	8.72 ± 2.45	7.28 ± 2.29	0.019 *
*RUNX3* site4	7.23 ± 1.51	6.45 ± 1.50	0.033 *
*RUNX3* site5	5.03 ± 0.82	4.59 ± 0.95	0.036 *
*RUNX3* site6	4.76 ± 1.55	4.31 ± 0.50	0.046 *
*RUNX3* site7	2.20 ± 0.93	1.85 ± 0.44	0.021 *
*RUNX3* site8	2.62 ± 1.27	2.06 ± 0.68	0.042 *
*RUNX3* site9	5.07 ± 2.06	4.10 ± 1.27	0.044 *
*RUNX3* Total	6.03 ± 1.91	5.05 ± 1.58	0.029 *

Mann–Whitney test was used here. * *p* < 0.05.

**Table 5:** Correlation between AFB1 exposure, liver function, and DNA methylation.

	AFB1 EDI (ng/kg·bw/d)	
	Correlation Coefficient	*p*
Liver function and lipid metabolism		
AST (U/L)	0.049	0.555
ALT (U/L)	0.014	0.861
ALP (U/L)	0.096	0.240
GGT (U/L)	0.111	0.197
TP (g/L)	−0.121	0.139
TBIL (μmol/L)	0.092	0.261
ALB (g/L)	0.053	0.526
*RUNX3* methylation (% 5mC)		
*RUNX3* site1	−0.071	0.409
*RUNX3* site2	−0.196	0.021 *
*RUNX3* site3	−0.109	0.202
*RUNX3* site4	−0.083	0.325
*RUNX3* site5	−0.040	0.638
*RUNX3* site6	0.006	0.943
*RUNX3* site7	−0.047	0.583
*RUNX3* site8	−0.192	0.024 *
*RUNX3* site9	−0.181	0.036 *
*RUNX3* Total	−0.078	0.353

Spearman correlation analysis was used here. * *p* < 0.05.

**Table 6:** Correlation between DNA methylation and liver function.

	*RUNX3* Site 2 Methylation		*RUNX3* Site 8 Methylation		*RUNX3* Site 9 Methylation	
	Correlation Coefficient	*p*	Correlation Coefficient	*p*	Correlation Coefficient	*p*
Liver function and lipid metabolism						
AST (U/L)	−0.060	0.483	0.082	0.337	0.023	0.792
ALT (U/L)	0.049	0.565	0.109	0.203	0.094	0.280
ALP (U/L)	−0.080	0.348	−0.135	0.116	−0.101	0.242
GGT (U/L)	0.097	0.273	0.206	0.019 *	0.203	0.024 *
TP (g/L)	0.063	0.460	0.058	0.500	0.047	0.585
TBIL (μmol/L)	−0.037	0.660	0.004	0.963	0.006	0.948
ALB (g/L)	−0.132	0.128	−0.103	0.240	−0.104	0.241

Spearman correlation analysis was used here. * *p* < 0.05.

## Data Availability

The data are not publicly available due to the privacy or ethical.

## Appendix A

**Table A1:** Stratified analysis.

	Low AFB1 Exposure (n = 75)	High AFB1 Exposure (n = 75)	*p*
	Mean ± SD	Mean ± SD	
*RUNX3* site1			
HBsAg+	4.11 ± 2.14	5.93 ± 3.78	0.197
HBsAg^−^	6.70 ± 3.48	6.13 ± 3.22	0.656
*p*	0.012 *	0.523	
*RUNX3* site2			
HBsAg+	8.61 ± 3.53	9.57 ± 2.99	0.400
HBsAg^−^	13.22 ± 5.73	11.03 ± 4.97	0.044 *
*p*	0.006 *	0.510	
*RUNX3* site3			
HBsAg+	6.60 ± 2.31	7.97 ± 2.17	0.165
HBsAg^−^	8.91 ± 2.59	8.52 ± 2.29	0.469
*p*	0.009 *	0.617	
*RUNX3* site4			
HBsAg+	5.85 ± 1.28	7.05 ± 1.53	0.089
HBsAg^−^	7.30 ± 1.52	7.16 ± 1.52	0.475
*p*	0.006 *	0.749	
*RUNX3* site5			
HBsAg+	4.11 ± 0.83	5.02 ± 0.86	0.061
HBsAg^−^	5.08 ± 0.74	4.98 ± 0.91	0.460
*p*	0.001 *	0.910	
*RUNX3* site6			
HBsAg+	4.03 ± 0.34	4.56 ± 0.49	0.020 *
HBsAg^−^	4.63 ± 0.61	4.89 ± 2.12	0.869
*p*	0.002 *	0.857	
*RUNX3* site7			
HBsAg+	1.57 ± 0.18	2.08 ± 0.47	0.006 *
HBsAg^−^	2.32 ± 1.16	2.08 ± 0.58	0.470
*p*	0.001 *	0.818	
*RUNX3* site8			
HBsAg+	1.67 ± 0.25	2.37 ± 0.77	0.010 *
HBsAg^−^	2.90 ± 1.43	2.30 ± 0.99	0.082
*p*	0.001 *	0.482	
*RUNX3* site9			
HBsAg+	3.42 ± 0.45	4.71 ± 1.47	0.022 *
HBsAg-	5.51 ± 2.35	4.58 ± 1.56	0.117
*p*	0.005 *	0.637	
*RUNX3* Total			
HBsAg+	4.55 ± 1.43	5.54 ± 1.63	0.190
HBsAg^−^	6.18 ± 2.04	5.86 ± 1.76	0.583
*p*	0.010 *	0.673	

Mann–Whitney test was used here. * *p* < 0.05.

**Table A2:** Correlation between AFB1 exposure, liver function, and DNA methylation in HBsAg^−^ population.

	AFB1 EDI (ng/kg·bw/d)	
	Correlation Coefficient	*p*
Liver function and lipid metabolism		
AST (U/L)	0.039	0.662
ALT (U/L)	−0.014	0.876
ALP (U/L)	0.115	0.199
GGT (U/L)	0.071	0.449
TP (g/L)	−0.127	0.154
TBIL (μmol/L)	0.141	0.114
ALB (g/L)	0.094	0.299
*RUNX3* methylation		
*RUNX3* site1	−0.192	0.030 *
*RUNX3* site2	−0.241	0.008 *
*RUNX3* site3	−0.207	0.019 *
*RUNX3* site4	−0.180	0.043 *
*RUNX3* site5	−0.139	0.118
*RUNX3* site6	−0.015	0.871
*RUNX3* site7	−0.156	0.080
*RUNX3* site8	−0.288	0.001 *
*RUNX3* site9	−0.295	0.001 *
*RUNX3* Total	−0.184	0.038 *

Spearman correlation analysis was used here. * *p* < 0.05.

**Table A3:** Correlation between DNA methylation and liver function in HBsAg^−^ population.

	*RUNX3* Site 1 Methylation		*RUNX3* Site 2 Methylation		*RUNX3* Site 3 Methylation		*RUNX3* Site 4 Methylation		*RUNX3* Site 8 Methylation		*RUNX3* Site 9 Methylation	
	Correlation Coefficient	*p*	Correlation Coefficient	*p*	Correlation Coefficient	*p*	Correlation Coefficient	*p*	Correlation Coefficient	*p*	Correlation Coefficient	*p*
Liver function and lipid metabolism												
AST (U/L)	0.016	0.857	−0.050	0.583	0.017	0.852	−0.002	0.984	0.021	0.819	0.035	0.696
ALT (U/L)	0.109	0.223	0.036	0.699	0.103	0.249	0.083	0.351	0.069	0.445	0.091	0.314
ALP (U/L)	−0.111	0.215	−0.130	0.156	−0.108	0.227	−0.151	0.090	−0.209	0.020 *	−0.192	0.032 *
GGT (U/L)	0.122	0.189	0.074	0.440	0.112	0.228	0.121	0.194	0.144	0.124	0.142	0.132
TP (g/L)	0.059	0.511	0.072	0.432	0.057	0.522	0.047	0.602	0.014	0.877	0.019	0.835
TBIL (μmol/L)	0.032	0.719	−0.024	0.795	0.026	0.769	0.056	0.534	0.052	0.568	0.070	0.437
ALB (g/L)	−0.114	0.210	−0.142	0.128	−0.141	0.121	−0.123	0.177	−0.157	0.086	−0.125	0.174

Spearman correlation analysis was used here. * *p* < 0.05.

**Table A4:** Factorial analysis of methylation levels of all nine CpG site of *RUNX3* with AFB1 exposure and HBV infection.

	HBsAg+	HBsAg^−^	HBV Main Effect		AFB1 Main Effect		HBV *AFB1	
	Mean ± SD	Mean ± SD	F	*p*	F	*p*	F	*p*
*RUNX3* site1								
Low AFB1 exposure	4.11 ± 2.14	6.70 ± 3.48	3.106	0.080 *	0.631	0.428	2.294	0.132
High AFB1 exposure	5.93 ± 3.78	6.13 ± 3.22						
*RUNX3* site2								
Low AFB1 exposure	8.61 ± 3.53	13.22 ± 5.73	5.696	0.018 *	0.232	0.631	1.536	0.217
High AFB1 exposure	9.57 ± 2.99	11.03 ± 4.97						
*RUNX3* site3								
Low AFB1 exposure	6.60 ± 2.31	8.91 ± 2.59	5.946	0.016 *	0.702	0.404	2.257	0.135
High AFB1 exposure	7.97 ± 2.17	8.52 ± 2.29						
*RUNX3* site4								
Low AFB1 exposure	5.85 ± 1.28	7.30 ± 1.52	4.606	0.034 *	2.163	0.144	3.424	0.066
High AFB1 exposure	7.05 ± 1.53	7.16 ± 1.52						
*RUNX3* site5								
Low AFB1 exposure	4.11 ± 0.83	5.08 ± 0.74	5.629	0.019 *	4.251	0.041 *	6.669	0.011 *
High AFB1 exposure	5.02 ± 0.86	4.98 ± 0.91						
*RUNX3* site6								
Low AFB1 exposure	4.03 ± 0.34	4.63 ± 0.61	1.799	0.182	1.337	0.249	0.160	0.690
High AFB1 exposure	4.56 ± 0.49	4.89 ± 2.12						
*RUNX3* site7								
Low AFB1 exposure	1.57 ± 0.18	2.32 ± 1.16	3.112	0.080 *	0.416	0.520	3.153	0.078
High AFB1 exposure	2.08 ± 0.47	2.08 ± 0.58						
*RUNX3* site8								
Low AFB1 exposure	1.67 ± 0.25	2.90 ± 1.43	4.145	0.044 *	0.032	0.858	5.165	0.025 *
High AFB1 exposure	2.37 ± 0.77	2.30 ± 0.99						
*RUNX3* site9								
Low AFB1 exposure	3.42 ± 0.45	5.51 ± 2.35	4.219	0.042 *	0.145	0.704	5.397	0.022 *
High AFB1 exposure	4.71 ± 1.47	4.58 ± 1.56						
*RUNX3* Total								
Low AFB1 exposure	4.55 ± 1.43	6.18 ± 2.04	4.642	0.033 *	0.553	0.458	2.122	0.147
High AFB1 exposure	5.54 ± 1.63	5.86 ± 1.76						

General linear model test was used here. * *p* < 0.05.
